# Interdisciplinary education affects student learning: a focus group study

**DOI:** 10.1186/s12909-023-04103-9

**Published:** 2023-03-18

**Authors:** Jessica Oudenampsen, Marjolein van de Pol, Nicole Blijlevens, Enny Das

**Affiliations:** 1 Department of Hematology, Radboudumc Medical Center, Geert Grooteplein Noord 21, 6525EZ Nijmegen, The Netherlands; 2 Department of Primary and Community Care, Radboudumc Medical Center, Nijmegen, The Netherlands; 3 Department of Hematology, Radboudumc Medical Center, Nijmegen, The Netherlands; 4 Centre for Language Studies, Radboud University, Nijmegen, The Netherlands

**Keywords:** Interdisciplinary education, Interdisciplinary approach, Higher education, Medical education, Communication education

## Abstract

**Background:**

In order to best prepare medical students for their increasingly complex future career, interdisciplinary higher education is swiftly gaining popularity. However, the implementation of interdisciplinary learning in medical education is challenging. The present study deepens the understanding of the challenges and opportunities inherent to the implementation of an interdisciplinary course. We elucidated the attitudes and beliefs of students participating in a newly developed interdisciplinary minor, in which students of medicine (MS) and communication and information sciences (CISS) were involved.

**Methods:**

We conducted four semi-structured focus group interviews, of which two were held before, and two were held after the course. Seven MS and six CISS participated voluntarily. A pre-arranged interview guide was used. The interviews were recorded and afterwards systematically analyzed with the ‘constant comparative analysis’ technique.

**Results:**

The focus group interviews revealed three differences in epistemics between students in terms of 1) curriculum content, 2) educational formats and 3) student’s competence perceptions. These factors influenced the way students evaluated themselves, each other and the interdisciplinary course.

**Conclusions:**

We conclude that factors that influence interdisciplinary learning are personal epistemics, individual learning preferences, and the synergy that is achieved throughout interdisciplinary learning. Organizing the dialogue among students of different disciplines could make students aware of inequalities, implicated biases and assigned status of different student groups. These empirical results are crucial to tailor interdisciplinary education to each specific discipline and to take interdisciplinary learning to a higher level of maturity.

**Supplementary Information:**

The online version contains supplementary material available at 10.1186/s12909-023-04103-9.

## Introduction

Considering how rapidly society is changing in almost all life domains, we will increasingly need to develop new skills, tools and techniques to solve complex challenges in our work and societal environment. These complex challenges are often influenced by multiple factors that are studied separately by different disciplines. A monodisciplinary approach solves some single aspects of complex problems, however to arrive at comprehensive solutions, integration of knowledge and skills from different disciplines and knowledge domains is necessary [1], for which an interdisciplinary environment is required. In such environments, such as Universities, the interdisciplinary approach is swiftly gaining popularity [2].

The development and implementation of interdisciplinary learning in higher education curricula, however, require specific conditions that can be challenging to fulfill, such as teachers skilled in team development and interdisciplinarity, and students who can think creatively outside their own realm [1]. The present research therefore aims to identify challenges and possible opportunities for interdisciplinary learning. We assessed educational outcomes as well as attitudes and beliefs of students participating in a newly developed interdisciplinary minor ‘Healthcare communication, management and organization’. Insights drawn from our data increase understanding of the practical effectuation of challenges and barriers in the specific disciplines of health- and communication sciences.

Interdisciplinary learning generates outcomes that differ from monodisciplinary learning outcomes. As interdisciplinary learning takes place in the overlap between disciplines, students are expected to synthesize and integrate not only abstract knowledge and theories used in different disciplines, but also the way the knowledge and theories are obtained, taught and used in these disciplines [3].

The most comprehensive learning outcome of interdisciplinary learning is defined as ‘interdisciplinary thinking’, i.e.,

“the capacity to integrate knowledge and modes of thinking in two or more disciplines or established areas of expertise to produce a cognitive advancement, such as explaining a phenomenon, solving a problem, or creating a product, in ways that would have been impossible or unlikely through single disciplinary means” [4].

As reflected by the growing number of established practices in interdisciplinary learning [2] and the wide variety of disciplines participating in the courses, e.g., courses integrating humanities, social and nature sciences [5], business, law and health [6] and media and teaching students [7], there is a general consensus that interdisciplinary teaching can support students to gradually develop these interdisciplinary thinking skills. According to this definition, interdisciplinary thinking can be considered a complex cognitive skill that consists of a number of subskills and learning outcomes [8]. Besides generating advances in cognitive ability [9], interdisciplinary learning can increase the ability to recognize bias, think critically and tolerate ambiguity [10–12].

Another outcome of interdisciplinary learning pertains to understanding the differences between disciplines, i.e., assumptions, ideologies and beliefs on which education in a discipline rests [13]. Moreover, boundary-crossing skills can be achieved such as the ability to change perspectives, to synthesize knowledge of different disciplines, and to cope with complexity. Interdisciplinary education also presents challenges, however, insofar as it does not occur spontaneously while attending interdisciplinary education; successful interdisciplinary learning depends on the (characteristics of) the disciplines, on teacher skills and on students’ characteristics as elaborated upon below.

Currently, research into interdisciplinary education is scarce [14]. The importance of interdisciplinary learning is often theorized, however these claims are seldom based on empirical evidence [6, 15] and a solid overview of established research and practices in interdisciplinary learning is lacking [14]. There is a slowly growing body of -predominantly theoretical- literature that has identified facilitators and barriers for interdisciplinary learning, but empirical findings are few and far between [1].

As established by mostly descriptive explorations in the past four decades, one factor possibly influencing interdisciplinary learning are the epistemics [3, 16–18]. Epistemics, the disciplinary ideas about what knowledge is and how to use and produce knowledge, are part of the culture of a discipline [3, 19]. Although the epistemics of a discipline are mainly implicit, the specificities of a discipline and the way in which it handles knowledge can be made explicit in interdisciplinary learning [20]. When made explicit, the principles and assumptions arising from these epistemics are different from those of the home discipline and are frequently hard to understand [19]. Consequently, they can possibly hamper the effectiveness of collaboration [21, 22], or can even provoke strong rejection with great underlying emotional convent, although the latter claim is not substantiated by (empirical) evidence [23]. In addition, differences in epistemics are thought to present even more severe hurdles when combining two completely distinct sciences such as health sciences and language sciences [24]. For example, evaluation research showed reduced initial preparedness for intellectual collaboration and a slower pace of collaborative activities when researchers represented a wide spectrum of disciplinary perspectives among team members [21].

Another factor influencing interdisciplinary learning is the ritualized manner of communication in different disciplines [18, 23]. The absence of a common language results in difficulties in adequate communication between practitioners of different disciplines, as showed by a review on team performance [17], and by an evaluation of an interdisciplinary course [25].

Perceptual barriers are a third factor relevant for interdisciplinary learning [16, 17, 25]. An important aspect of these perceptual barriers are competence perceptions many scientists have about their own and other disciplines. For example, survey measures showed that social scientists are believed to have lower intelligence than natural scientists, an assumption also reflected in discussions on the hierarchy of sciences [25]. Likewise, empirical research in health sciences showed that more than half of the research participants coming from social sciences altered their research to achieve some level of legitimacy in the eyes of their medical colleagues [16, 26]. The value of the science to society can also influence perceptions of the discipline. For example, a profession is found to have value if it is considered worthwhile to engage in and fund [27]. If such perceptual tensions and arrogance about being from a ‘superior’ discipline or lack of respect exist, this will affect the level and manner of perception and integration in interdisciplinary education [28]. Unfortunately, research on the prevalence of perceptual barriers in interdisciplinary education and in different disciplines is scarce and the existing evidence remains unclear.

Another factor influencing interdisciplinary learning refers to the importance of teacher teams and their professional interdisciplinary skills. Teachers should be able to facilitate the necessary understanding of each other’s disciplines, as well as the integration of the different disciplines. Furthermore, they need to be able to manifest a safe environment where students feel free to find their own way into interdisciplinarity [15].

Next to the abovementioned structural barriers, personal characteristics seem to affect the learning outcomes. As established in previous research [1, 17, 29], the necessary personal characteristics for enabling interdisciplinary thinking are curiosity, respect, and openness. In addition, patience, diligence, and self-regulation are described as essential characteristics for enabling the production of a cognitive advancement in interdisciplinary learning. Moreover, gender is found to be a predictive factor, with the female gender being significantly more receptive to interdisciplinary collaboration [25]. More in general, perceptions of warmth are also considered to influence collaboration between disciplines (as a measure of friendliness, trustworthiness, empathy and kindness) [25].

Overall, empirical evidence on facilitators and barriers for interdisciplinary learning remains scarce and limited to interdisciplinarity in research and teams, rather than education at the academic level. The present study will provide deeper insights in students’ perceptions towards interdisciplinary learning, their own and other disciplines. This will establish a deeper understanding of methodological and perceptual factors influencing interdisciplinary learning, as is crucial to change existing barriers into opportunities and increase the potential of interdisciplinary learning.

## Methods

A qualitative focus group study was conducted to investigate interdisciplinary learning and collaboration from the views of both Communication and Information Sciences students (CISS) and Medicine students (MS). Data were obtained during an interdisciplinary course called ‘Healthcare communication, management and organization’, held from February till May 2020 at the Radboud University in Nijmegen, Netherlands.

### Setting

#### Participants

Study participants were all undergraduate CISS and MS that participated in the interdisciplinary course. To participate in the course, students had to be at least in their third year of their BA program. A total of 22 CISS, for whom the course was mandatory, were enrolled. For MS this course was part of their elective program. Students were able to receive information about the course through information guides and information meetings. A total of seven MS participated.

#### Interdisciplinary course

During the eight-week interdisciplinary course in healthcare communication, management and organization, students worked together in interdisciplinary teams dealing with complex, ‘*real world problems’*, concerning communication in healthcare. Interdisciplinary problems, such as a scientific-linguistic issues in doctor-patient conversations and crisis management, were addressed during theoretical and practical education. Students had to fulfill group and individual assignments about the presented problems. Students from the different knowledge domains brought together their own expertise and applied these in the context of the others. For a more detailed description of the minor, including intended learning outcomes and assessments, see Additional file 1.

### Study design

#### Focus group interviews

Four semi-structured focus groups were conducted to examine in-depth the elements that support interdisciplinary thinking. The focus groups draw on group interaction, which encouraged participants to explore and clarify their views in more detail. As homogeneity in focus groups is considered essential for group interaction and dynamics, study background was used to ensure some degree of homogeneity [30], i.e., during the focus groups only peers from participant’s own study were present. To enable all participants to contribute substantially to the discussion, the groups were kept relatively small, yet large enough to stimulate discussion and produce new insights. All seven MS participating in the course agreed to participate in the study. To create comparable groups, the same number of CISS were randomly invited to participate in the study. Six CISS participated.

During the study, four focus group sessions were held. Two focus groups (one consisting of CISS and one consisting of MS) were held before the start of the course in February 2020 and therefore can be considered as baseline interviews. The other two sessions took place at the end of the course, in May 2020. Discussions in the focus groups lasted about one hour. Students were ascertained that the content of the interviews would not affect their grades.

In order to focus on the possible facilitators and barriers for interdisciplinary learning, the questions posed to the participants were pre-arranged, as provided partially and as way of example in Table 1 and in detail in Additional file 2. Questions in the first sessions focused on four themes; 1) Expectations for learning outcomes, 2) Expectations for the collaboration with other students, 3) Prejudices and perceptual tensions towards the other students, and 4)Competence perceptions of students of their own discipline vs. the other discipline. The same four themes were used in the second interviews with students, although the focus was shifted to the evaluations of the given subjects rather than the expectations.

**Table 1:** Examples of interview questions

-What expectations do you have, both practically and socially, about the collaboration with the students from the other discipline?
- How do you define our own expertise within your field? (When asked to explain; What are you particularly good at?) During the first interview, you mentioned a number of expectations about the learning effects of this minor. Did these expectations come true?
- Which positive preconceptions about the students from the other discipline proved true, and which ones did not?

The focus group interview moderator was an independent researcher with experience in moderating focus groups; one of the authors (JO) observed the interviews and made field notes. The focus group moderator had no relationship with the students. The focus group interviews were audio-taped and transcribed verbatim.

### Data analysis

The transcripts were analyzed, using the constant comparative analysis technique [31] to first interpret the data qualitatively whilst secondly systematically looking at causality between the variables. Coding followed a three-part process [31]: 1) Open coding of all of the interviews by one of the researchers was used to develop an initial template of codes. 2) Independently open coding by two researchers followed by identifying focal themes. 3) Interpretations, disagreements and doubts about focal themes were discussed among the research team. Through constant further reducing and recoding during multiple sessions with the research team, final focal themes were identified. This was followed by selective and systematic coding by one researcher, using the core themes and modified code book. Analysis processing was supported by Atlas.ti v8.4.20 software.

### Ethical considerations

This study is part of the quality evaluation cycle of the course. The research participants are not subjected to acts that are subject to the WMO (Medical Research Involving Human Subjects Act) and they are not subject to any conduct that is subject to the WMO. On this basis, the Medical Faculty Science Committee of the Radboud University Medical Center (COMOS) declared that the research does not fall under the WMO. The research of this paper therefore does not require a positive judgment from the CMO region Arnhem-Nijmegen or any other recognized medical-ethical review committee. For quality reasons all research performed at our university undergoes study protocol review by our research board/licensing committee (COMOS). The study protocol was approved by COMOS in January 2020. All research that involved human subjects was conducted in accordance with the Declaration of Helsinki.

## Results

The codes and themes extracted from the focus group interviews are showed in Additional file 3. We identified three key concepts with inter-related effects on interdisciplinary learning after analysis of the focus group interviews with the different student groups. All concepts occurred in all of the four focus groups, and thus represent the most common topics. The concepts are presented below in more detail, and pertain to differences in epistemics in terms of 1) curriculum content, 2) educational format, and 3) competence perceptions. Concepts are illustrated with quotations from the participants (MS = Medicine student, CISS = CIS student).

### Epistemics - curriculum content

**Fig. 1:**
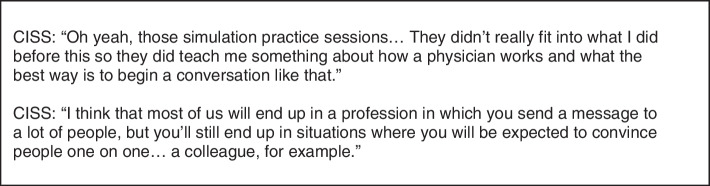
Quotes concerning epistemics – curriculum content [1]

Both groups of students mentioned that CISS have a broad perspective on epistemics, whereas medicine students have a more focused view on epistemics. CISS were interested in multiple different contexts. Students mentioned that CISS are used to looking at multiple levels of knowledge and zooming in-and out on theories. Moreover, students mentioned that CISS are used to learning about and working with statistics, data and analyses. The immersion into the unknown, within the new context of health sciences, motivated the CISS to learn. CISS mentioned valuable learning outcomes, even when theory or practice during the minor was not directly applicable in their study program or future professional career. (See Fig. 1 for supportive quotes).

**Fig. 2:**
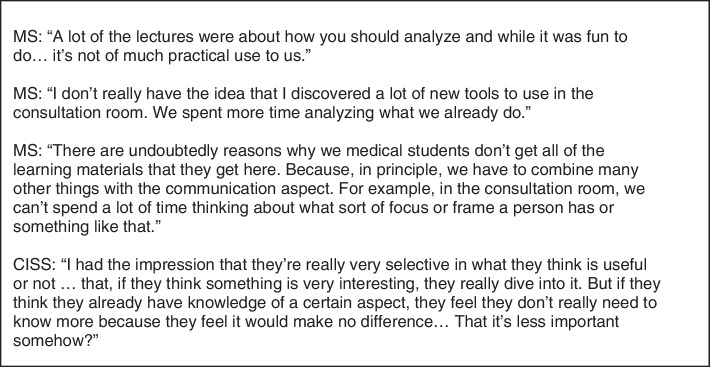
Quotes concerning epistemics – curriculum content [2]

Both student groups also mentioned that, in contrast, MS have a more focused view on epistemics, resulting in quite specific interests (Fig. 2). CISS stated that the selective interests of medicine students resulted in less motivation to learn about communication beforehand. Moreover, CISS mentioned that due to their specific interests focused on becoming a doctor, MS appeared to value only knowledge that is directly applicable in doctor-patient conversations. When not directly applicable in healthcare practice, the knowledge was regarded as ‘nice to know’ but did not move beyond that. For example, when specifically asked about scientific skills, MS said they learned to analyze and code texts in a scientific way, however they did not seem to gather being able to code and analyze texts under ‘learning outcomes’ because the knowledge was not directly applicable in healthcare conversations. MS mentioned they appreciated less the interdisciplinary aspect of the education, and perceived fewer aspects of the course as useful learning outcomes compared to CISS.

### Epistemics—educational format

**Fig. 3:**
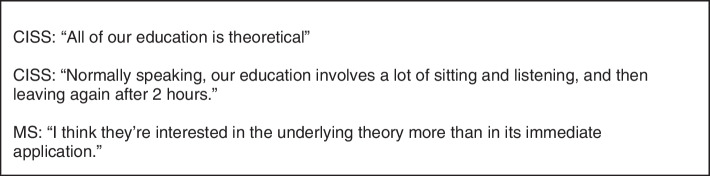
Quotes concerning epistemics – educational format [1]

**Fig. 4:**
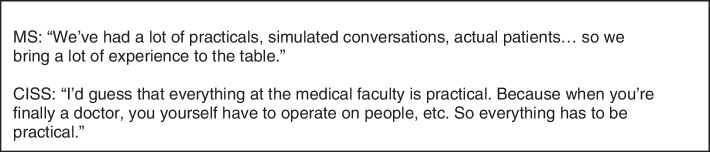
Quotes concerning epistemics – educational format [2]

CISS were used to theoretical education (lectures, reading and paper assignments), whereas MS prefer the practical education they normally have (simulation practice, practice related assignments, contact with patients). See Fig. 3 for quotes about the educational format for CISS. Figure 4 shows quotes about the educational format for MS.

**Fig. 5:**
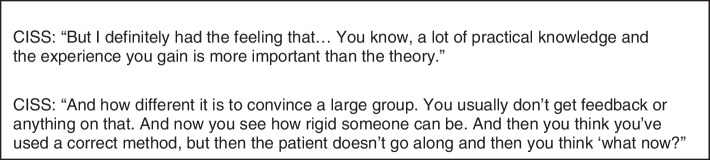
Quotes concerning epistemics – educational format [3]

CISS had previously acquired a lot of theoretical knowledge, which led to this minor feeling like a welcome change, an invitation to apply their knowledge in real life, to learn things from personal, practical, experience. For example, through the real-life practice, they became aware of differences between face to face communication (with immediate feedback from someone’s facial expressions) and mass-mediated communication (without receiving immediate feedback). The teaching- and assessment methods in this course were different from the methods CISS were used toas is reflected in the quotes in Fig. 5.

In contrast, MS regarded this theory as a hurdle. Real learning in their opinion takes place during practice based education and experience. Therefore, MS felt that the theoretical education in this minor was of little initial relevance for them. 

**Fig. 6:**
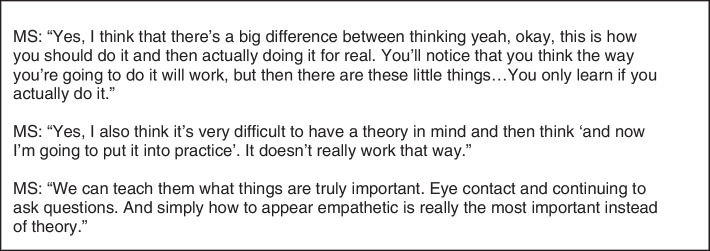
Quotes concerning epistemics – educational format [4]

Related to this, when asked afterwards, MS felt like the theoretical education they had during this minor, was less relevant than the skills they had previously acquired during their study. See Fig. 6 for supportive quotes of MS.

### Students’ competence Perceptions

**Fig. 7:**
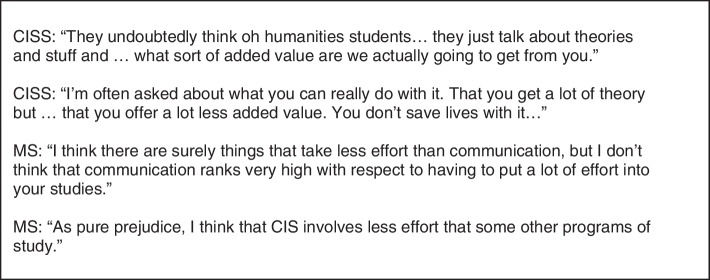
Quotes concerning students’ competence perceptions [1]

CISS voiced a clear opinion about their own study and status. CISS felt uncertain about the status of their own study, as reflected in quotes like ‘other students think we offer less added value’, ‘other students think we just learn to chat and present’ and ‘our study is seen as a study for fun’ (Fig. 7). CISS mentioned just three competence perceptions they assigned to themselves: Being analytical, their ability to convince and influence and their ability to communicate with organizations. Their self-confidence was further weakened by the perceptions that MS reported about CISS, MS said they didn’t learn anything from CISS and collaboration with CISS was difficult.

In contrast, MS were convinced about their own expertise and competencies, as well as about the high status of their study program. Also, during the course, MS felt strengthened in their perceptions about the own expertise, for example by comparing the doctor-patient conversations of themselves and the communication students’ conversations.

**Fig. 8:**
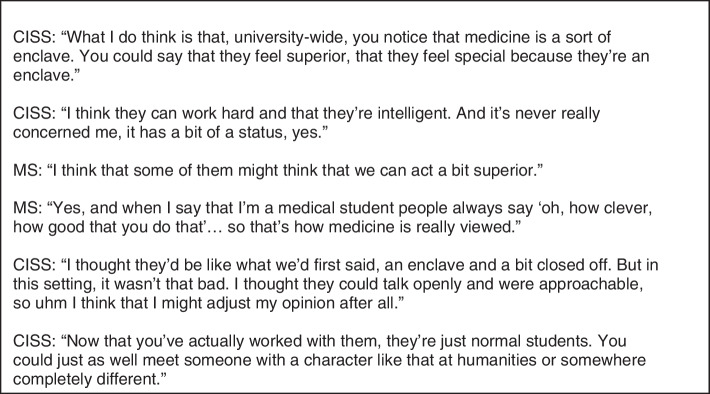
Quotes concerning students’ competence perceptions [2]

Before the start of the minor, CISS assumed that MS would be arrogant and behave in line with their high status. As CISS repeatedly mentioned, they looked up to the knowledge and status of MS. However, CISS were positively surprised by the attitude of- and collaboration with MS. For example, prejudices they mentioned like arrogance and being an enclave turned out not to be true and collaboration went very smoothly and effectively (Fig. 8).

## Discussion

This is one of the first empirical studies about perceived methodological, theoretical and perceptual barriers and facilitators for interdisciplinary learning. The findings confirm the premise that disciplinary epistemics can be a barrier to interdisciplinary learning in multiple ways. Specifically, we found differences in what students’ assume as knowledge that is of value and in assumptions about how to produce knowledge through education. These epistemics influenced the way students evaluated the education. Furthermore, we found differences in how students evaluated themselves and each other. Our study empirically substantiates existing theoretical frameworks [1, 17, 19, 22] and demonstrates that multidimensional factors concerning epistemics and different academic cultures play a role in the effectiveness of interdisciplinary learning.

### The value of ‘hard’ and ‘soft’ knowledge

The focus groups revealed a difference between disciplines in student’s personal epistemics concerning the cognitive dimension. CISS had a general orientation, which prompted the motivation to broaden their knowledge and skills, including knowledge from another discipline. In contrast, MS had a narrower view on what constitutes important knowledge. They were highly motivated to learn in-depth concepts directly pertinent to the healthcare profession, but hardly motivated to learn from communication students and about general academic skills such as scientific reading, analyzing texts and analyzing study results. It appeared that cognitive epistemics hindered interdisciplinary learning for MS in particular. Our findings provide empirical evidence for previous theoretical frameworks. As described by several authors [32–34], at the beginning of their studies, student’s personal epistemology in separate disciplines is rather similar. Differences in students’ personal epistemology appear, as they progress in their study and are immersed in the discipline specific culture. As a result, students’ personal epistemics are derived from the epistemics of the discipline they are studying.

Comparison of the personal epistemics of the students in our study with previous theoretical frameworks confirms that the cognitive dimension divides disciplines into hard and soft, pure and applied sciences [35, 36]. As CIS can be considered a soft discipline [3, 35, 36], knowledge is typically gained through a broad command of intellectual ideas, through creativity in thinking and fluency of expression. Career prospects are seen as open-ended because the curriculum is based on generalizable skills [37, 38]. General degrees are found to teach many kinds of courses without a clear orientation, so students are prepared for general qualifications for many kinds of jobs. Also, the shift from simple to multiple perspectives is found to be more common in humanities and social sciences in comparison to the hard sciences in which a shift requiring a more developed level of expertise occurred more often [34, 36, 39].

The hard sciences (as medicine can be considered), whether pure or applied, are known to hold a prominent place for facts, principles, quantitative methods, classification and categorization in the acquisition of knowledge [33, 40]. The intellectual skills developed during the study are predominantly specific, rather than generalizable outside the disciplinary context [34, 40]. This is also in line with findings from Lonka & Lindblom-Ylänne [41], who reported that medical undergraduate students were more likely to express professional orientation. Medical students appeared to be interested in active professional development instead of academic theoretical questions, and mostly directly applicable information was appreciated. These factors constitute a possible obstacle to their academic development. The current findings suggest that the above mentioned epistemic differences between the hard and soft sciences may hamper the outcomes of interdisciplinary learning, especially for the hard science students.

Interestingly, our findings reveal a paradox: Interdisciplinary learning is intended to facilitate boundary crossing and perspective change, but students who already possess the broadest perspective are also the most willing to learn about other perspectives and vice versa. This raises the question of how to cultivate a preparedness for learning from other perspectives, and thus for interdisciplinary learning, particularly among students with narrow perspectives (one might say; among the students who could benefit most from interdisciplinary learning). Our findings suggest that it is interdisciplinary learning itself that increases the preparedness for interdisciplinary learning, because experiencing a change of perspectives for the first time creates a willingness for further learning about other perspectives. This paradox has implications for interdisciplinary learning: it should be offered gradually and tailored to each specific discipline. At least our empirical results establish the need to pay sufficient attention to students’ epistemological development and their conceptions of learning during implementation and instruction of interdisciplinary education. Future research may focus on possible ways of offering interdisciplinary learning gradually. Furthermore, future research is suggested vis-à-vis exploring the best ways to break the vicious circle mentioned above, by exploring implicit biases and preferences of students from different disciplines. For example, by making different combinations of disciplines, i.e. two disciplines characterized by ‘hard sciences’ vs. combining the hard and the soft sciences.

### Differences in learning preferences

The results of this study showed differences in students’ preferences with regard to educational formats. CISS valued both in-depth theoretical education, as well as the practical education. They believed the practical education to be an addition to their theoretical knowledge, whereas for MS the theoretical education was considered less relevant than the skills they had already acquired during previous practice-based education. Although the findings about differences in personal preferences to receive information are consistent with previous evidence [42], recently generated evidence suggests that these personal preferences are on a continuous spectrum and could not be distinguished in different groups or (dichotomous) learning styles [43]. So although our results reflect a distinction in preferences between disciplines, it could only be suggested that these differences exist because students became accustomed to educational formats in a given discipline, rather than these preferences are based on a neuroscientific difference in learning styles of students from different disciplines. Nevertheless, our results showed that encountering new educational formats, different from what students in a given discipline are used to, whether or not they fit students’ preferences, do affect the appreciation of the interdisciplinary education. This indicates that sufficient attention should be paid to the educational formats and the context in which interdisciplinary learning takes place to prevent teaching one discipline more appropriately than another.

### Students’ competence perceptions

In this study, we found that intellectual value judgements and inequality between students from different disciplines may hinder interdisciplinary learning. MS were self-confident about their own expertise and their competencies, while CISS were hesitant about the value of their knowledge for medicine students. As a result, MS were, in contrast with CISS, not convinced about the synergy between both disciplines. These results supports evidence from previous research practices about existing differences in the values assigned to disciplines [1, 16–18, 25]. However, interdisciplinary learning requires a synergistic, meaningful combination of different perspectives. In addition, both disciplinary groups have to value this combination to make interdisciplinary learning successful [25]. Moreover, previous research suggests that an unwillingness to elucidate these differences contributes to a broader sense of reticence or reserve among students [44]. Hence, when the dialogue about these differences does not occur, differences will possibly become obstacles. However, when students are able to identify and dialogue about these differences, then these differences are able to become accelerators for generating valuable interdisciplinary knowledge [44]. In this regard, not only epistemics seem to play a role in the effectiveness, but also the assigned status and underlying thoughts about one’s own expertise and the expertise of others. This suggests that attitude, e.g., respect and openness, should be part of the definition of interdisciplinary thinking [4]. To be able to change these barriers into opportunities for interdisciplinary learning, further research is needed to identify these inequalities between specific disciplines and the factors contributing to the emergence of inequality, for example factors such as motivation, implicit biases and thoughts about other students from different disciplines. Future research could also apply other research methods to gain insights into how to manage diversity in groups, e.g. observational methods when interdisciplinary groups are working on real life problems.

### Strengths and weaknesses

The findings in this empirical study contribute to the existing knowledge of barriers and opportunities in interdisciplinary learning by offering valuable insights into the practical consequences of epistemic differences between disciplines. During the study, interdisciplinary learning with the combination of MS and CISS was investigated. This study is the first empirical investigation and testing of the theoretical frameworks about epistemics and barriers in an interdisciplinary learning environment concerning healthcare communication. The results complement previous theories, which were mostly composed independent of participating disciplines, with empirical findings. Although our results are based on a specific course, the results provide a deeper, analytical generalizable understanding of factors influencing interdisciplinary learning. The practical implications provide a basis for improving interdisciplinary learning. Nevertheless, future research should further verify to what extent all observed findings apply to interdisciplinary learning that involves different disciplines.

Being limited to a fixed time slot (the end of the course), this study was unable to evaluate possible long-term effects of interdisciplinary learning. Students may not experience the benefits of what they have learned until later as they progress through their studies. Many learning outcomes of interdisciplinary learning can be traced back to self-awareness and insights and those are often processes that develop gradually. Consequently, the question is whether different or more results, for example more insights into the value of perspective changing, would be found if additional interviews were held later in time (e.g., a year later). A second limitation is that selection bias can not be ruled out because the medicine students voluntarily elected this course and may thus have had (implicit) bias regarding the effects of interdisciplinary learning. A question that was not addressed in this study is whether students would have achieved the same learning outcomes following the same course without being confronted with students from other disciplines and thus working monodisciplinarily.

## Conclusions and implications

In conclusion, the results of our study show that: 1) Regardless which disciplines are combined in interdisciplinary learning, it is important to be aware in advance about the epistemics and individual learning preferences that students have, in order to prepare them for interdisciplinary educational courses ensuring a meaningful combination and effective learning, 2) If the scope of the epistemics is clearly very divergent, it seems important to emphasize what synergy can be achieved throughout education, so that students know where and what they can learn from each other. These main findings underline an important role for conducting the dialogue between students from different disciplines in promoting interdisciplinary learning. Furthermore 3), It is necessary to be aware of the assigned status of different student groups, in order to achieve equality as well as to prevent implicit bias and disciplinary snobbery.

## Supplementary Information

**Additional file 1.** Structure and learning objectives Minor.

**Additional file 2.** Focusgroup interviews 2020.

**Additional file 3.** Codebook.

## Data Availability

The dataset supporting the conclusions of this article is available in the DANS repository, 10.17026/dans-zpq-7a3c.

## Abbreviations

CISS: Communication and information student
MS: Medical students
WMO: Medical Research Involving Human Subjects Act
COMOS: Medical Faculty Science Committee of the Radboud University Medical Center
