# Supplementary material for: Interdisciplinary education affects student learning: a focus group study
Source: BMC Med Educ. 2023 Mar 18;23:169. doi: 10.1186/s12909-023-04103-9 (PMC10024401; doi:10.1186/s12909-023-04103-9)
Supplement: Supplementary file 1 — Additional file 1. Structure and learning objectives Minor. [file 12909_2023_4103_MOESM1_ESM.docx]

Structure and learning objectives Minor

# Structure of the minor

The minor Healthcare communication, management and organization consists of three parts:

1. Practice based communicative innovations: A communication part in which direct and indirect communication with (groups of) patients (an essential clinical skill), colleagues, possibly in managerial and or administrative positions, and with society is discussed (every Monday and Wednesday);
2. Healthcare management and organization: A part in which patient care in a management and organizational perspective is discussed (every Thursday and Friday);
3. Business case communicative innovation: A section integrating the communication part and the management and organization part (week 8,9 and 10 of the minor).

Component (I) and (II) are divided into several topics. Linking clinic features to organization provides an opportunity to highlight how healthcare has changed at the national, regional, and local levels and the consequences this has had for patient care.
In addition, much has changed in communication with patients over the past decades resulting in a system based on shared decision making. All this also has consequences for social discussions such as the funding of care. Scientific literature together with these social developments will be the basis for dealing with a future perspective for management, organization and communication in healthcare.
All components of the minor are open to undergraduate students from three faculties, namely the medical, management, and literature faculties. For medical students, the three components together form a minor (of 14EC); for students of the management and literature faculty, the separate components (I) and (II) constitute (elective) courses.

# Overall learning traject

For medical students, the entire minor covers 10 weeks. Component (I) and component (II) are covered in the first 7 weeks. Component (III) covers the last three weeks of the minor. Every Monday and Wednesday is spent on component (I). Every Thursday and Friday is spent on component (II). Every Tuesday there is time for self-study for all components and preparing for the business case assignment.

# Learning objectives

The learning objectives are divided among the three different components.

*Learning objectives practice-oriented communicative interventions – linguistic lab*

You can describe the various aspects of communication, both with respect to style and form factors, which are important for therapy compliance, apply them in your own doctor-patient conversations and afterwards (quantitatively and qualitatively) analyze them. In the module Linguistic lab in the consulting room you will demonstrate the extent to which you have mastered the following learning objectives:

|  | **Learning objective** |
| --- | --- |
| 1 | You can describe different behavioural patterns and the influence on conversation styles and deduce your own characteristics and style(s) from this. |
| 2 | You can apply the knowledge gained through analysis about factors that contribute to therapy adherence in doctor-patient conversations and reflect on the application of these factors. |
| 3 | You can describe the different styles and forms that can be used in communication and influence adherence. |
| 4 | You can quantify linguistic expressions based on knowledge of styles and forms in communication and knowledge of coding of conversations. You can process these results in data files, and analyze and report the data according to (scientific) guidelines. |
| 5 | You can recognize and name the differences in background knowledge, different aspects and contexts of the students of the other faculty and thereby learn and collaborate in an optimal way in interdisciplinary education. |
| 6. | You can provide a reasoned case for the impact of the communicative intervention on users, stakeholders and society. |
| 7 | You can scientifically substantiate the choices made in the improvement report with the help of literature in both the medical and linguistic domain. |

*Learning objectives practical communicative innovations – interactive case/ crisis communication*You can develop or improve an intervention in a team context, based on the analysis of context, stakeholders, impact and scientific literature and using the various theories from the fields of medicine and communication and information sciences. You incorporate your knowledge gained on the internship-day into an improvement report regarding one of the case solutions. With this component, you demonstrate mastery of the following learning objectives:

|  | **Learning objective** |
| --- | --- |
| 1. | You can provide a reasoned argument for the impact of the communicative intervention on users, stakeholders and society. |
| 2. | You can scientifically substantiate the choices made in the improvement report using literature in both the medical and linguistic domains. |
| 3. | You can recognize and analyze the differences in background knowledge, different aspects and different contexts of the students of the other faculty and thereby learn and collaborate in an optimal way in interdisciplinary education. |

*Learning objectives healthcare management and organization*

**Module 1 – Decision-making in healthcare**

|  | **After this module students are able to:** |
| --- | --- |
| 1.1 | Explain the economic principles of healthcare decisions |
| 1.2 | Know typical mistakes in human decision making |
| 1.3 | Apply these decision heuristics to healthcare problems |
| 1.4 | Know how to evaluate the benefits and costs of healthcare interventions |

**Module 2 – Redesigning and changing healthcare organizations**

|  | **After this module students are able to:** |
| --- | --- |
| 2.1 | Explain how different societal challenges can be related to the design of organizations |
| 2.2 | Explain the key features of organization design |
| 2.3 | Explain the 3-D model of organizational change |
| 2.4 | Assess the organization design of a hospital |
| 2.5 | Write a plan of improvement and suggest ways to change the hospital |

**Module 3 – innovation, patenting and (expensive) medicines**

|  | **After this module students are able to:** |
| --- | --- |
| 3.1 | Explain different economic logics of price formation |
| 3.2 | Understand how these different logics do (not) operate in the pharmaceutical industry |
| 3.3 | Explain the economic rationale for intellectual property right protection (IPR) |
| 3.4 | Understand the business models underlying expensive medicines |

**Module 4 – network care**

|  | **After this module students are able to:** |
| --- | --- |
| 4.1 | Explain different forms of networks and network governance |
| 4.2 | Explain the key determinants of network success/failure |
| 4.3 | Assess real-life healthcare networks on their goals, structure and governance |
| 4.4 | Write a plan of improvement for a real-life healthcare network |

*Learning objectives business case innovative communication*

|  | **Learning objective** |
| --- | --- |
| 1. | You can identify and analyze communicative, economic, organizational and management challenges in healthcare and use them to formulate a recommendation for an intervention in the analyzed healthcare process. |
| 2. | You can describe and analyze the risks that prevent successful implementation or scale-up of your intervention. |
