# Supplementary material for: Interdisciplinary education affects student learning: a focus group study
Source: BMC Med Educ. 2023 Mar 18;23:169. doi: 10.1186/s12909-023-04103-9 (PMC10024401; doi:10.1186/s12909-023-04103-9)
Supplement: Supplementary file 2 — Additional file 2. Focusgroup interviews 2020. [file 12909_2023_4103_MOESM2_ESM.docx]

**Focusgroup interviews 2020**

Global content:
1^st^ focus group interviews (3^rd^ and 4^th^ of February), consist of four parts:
* Expectations of the minor (both practical and learning effects)
* Expectations of working with other students (both practical and learning effects)
* Prejudices about the other students
* How do you define your own expertise and what can students from the other discipline learn from you?

2^nd^ focus group interviews (17^th^ of February), consist of four parts:
* Evaluation of the minor based on expectations, practical and learning effects
* Evaluation of the cooperation with other students
* Prejudices about the other students
* How do you define your own expertise and what learned students from the other discipline from you?

Explicit content 1^st^ focus group interviews:
* Introduction:
- Welcoming;
- Explanation of the purpose of this interview (measurement of attitudes and learning outcomes and get insights for improving the minor for next year);
- Explanation of the structure of the interview (interviewer steers the conversation, some questions are fixed but there is also the opportunity for own questions and answers / own interpretation);
- Explanation of confidentiality of this interview results (data will remain confidential, will not affect the results obtained in the minor, data will be stored anonymously and securely).

* Questions:
- Expectations of the minor
 > Based on the information you received on beforehand and on the first day of this minor, what expectations do you have of what you will be doing during this minor?
 > What expectations do you have, again based on the information you received on beforehand, of what you are going to learn during this minor?

- Expectations about collaboration
 > What expectations do you have, both practically and socially, about the collaboration with the students from the other discipline?
> What do you expect to learn from the students from the other discipline in particular?

- Prejudices about other students
> Have you ever worked together with students of another discipline and the discipline involved in this minor in particular?
> What positive preconceptions do you have about the students from the other discipline?
> What negative preconceptions do you have about the students from the other discipline?
> In general, are you positive or negative about the students of the other discipline?

- Defining your own expertise
> How do you define our own expertise within your field? (When asked to explain; What are you particularly good at?)
> What can students from the other discipline learn from you in particular?
> What might the students from the other discipline see in you as negative characteristics?
> What might the students from the other discipline see in you as positive characteristics?

*Closure
> Any remaining comments that have not been addressed?
> Explaining follow up: At the end of the minor, another focus group interview will be conducted following the same procedure.

Explicit content 2^nd^ focus group interviews:
*introduction:
- Welcoming;
- Explanation of the purpose of this interview (measurement of attitudes and learning outcomes and get insights for improving the minor for next year);
- Explanation of the structure of the interview (interviewer steers the conversation, some questions are fixed but there is also the opportunity for own questions and answers / own interpretation);
- Explanation of confidentiality of this interview results (data will remain confidential, will not affect the results obtained in the minor, data will be stored anonymously and securely).

*Questions:
- Expectations of the minor:
> During the first interview, you mentioned a number of expectations of the minor. Have these expectations been met in terms of practicality and content?
> During the first interview, you mentioned a number of expectations about the learning effects of this minor. Did these expectations come true?
> Did you learn something during this minor that you did not expect to learn on beforehand?

- Expectations about collaboration:
> During the first interview, you expressed expectations about the collaboration with the students from the other discipline, have these expectations been fulfilled?
> Follow-up question: Why yes/no?
> What in particular did you learn from the students from the other discipline?

- Prejudices about other students:
> Which positive preconceptions about the students from the other discipline proved true, and which ones did not?
> What negative preconceptions about the students from the other discipline proved true, and which ones did not?
> Are there any other judgements you have about the students from the other discipline in retrospect?
> In general, are you positive or negative about the students from the other discipline?

- Defining your own expertise:
> How do you define our own expertise within your field? (When asked to explain; What are you particularly good at?)
> What in particular students from the other discipline have been able to learn from you?
> What did the students from the other discipline see in you as negative characteristics?
> What did the students from the other discipline see in you as positive characteristics?

- Interdisciplinary learning:
> In what ways has working with students from another discipline proved different from working with students from your own discipline?
> In what ways did you learn something about or through interdisciplinary learning?

*Closure
> Any remaining questions that have not been addressed?
