# Supplementary material for: Interdisciplinary education affects student learning: a focus group study
Source: BMC Med Educ. 2023 Mar 18;23:169. doi: 10.1186/s12909-023-04103-9 (PMC10024401; doi:10.1186/s12909-023-04103-9)
Supplement: Supplementary file 3 — Additional file 3. Codebook. [file 12909_2023_4103_MOESM3_ESM.docx]

**Appendix C - Codebook**

| **Theme** | **Code Group** | **Codes** |
| --- | --- | --- |
| Epistemics – curriculum content | Learning goals | -Learn from each other (in terms of subject matter) -Getting to know each others educational formats  -Doctor-patient communication  -Raise awareness  -Acquiring broader knowledge  -Combining knowledge  -Learn to collaborate  -Learning theory concerning practice  -Applying theory in practice  -Immersing in a specific context |
|  | Interdisciplinary Thinking | -Different view on problems -Awareness within other context  -Seeing the bigger picture -Insight into one’s own expertise  -Gaining insights  -Education about interdisciplinary learning  -Added value of other discipline -Differences in educational forms -Differences between disciplines |
| Epistemics – educational format | The design of the course | -Background knowledge  -Educational output  -Educational formats  -Organisation of the course  -Reflection  -Assessment |
|  | Teaching/ Teachers | -Consensus among teachers -Sharing of own experiences -Dynamically |
|  | Learning conditions | -Connection of educational components -Different physical environment -Multiple studies -Majority of CIS students -Emphasis on gaining experience  -Educational formats -Receiving feedback  -Compulsory education -Limited time for learning theory -Hospital setting |
|  | Teaching methods | -Active education -Case study -Real life practice  -Collaboration |
|  | Previous experiences | -Other educational formats  -Other study -Healthcare communication -Experience with doctor-patient conversations  -Interfaculty minor -Collaboration with students with another student |
| Students’ competence perceptions | Collaboration | -Different study attitude -Bring out the best in each other -Context dependent -Complement each other -Good communication -Group dynamics -Motivation -Personal differences -Social interaction -Difference in approach -Differences in knowledge(level) |
|  | Group dynamics | -Other interests -Sitting apart -Physical distance -Group dynamics -Majority of CIS students -Stand up for oneself -Social interaction -Prejudices about each other |
|  | Student characteristics | -Active attitude -Curious -Letting each other talk -Ask each other questions -Want to get to know each other -Enthusiastic -Motivation -Open attitude -Stand up for oneself -Selectively interested |
|  | Prejudices and judgements | -Prejudices about each other -Prejudices about medicine students -Prejudices about CIS students -Self-judgement of medicine students -Self-judgement of CIS students |
|  | Prejudices about medicine students | -Active attitude -Analytical -Competitive -Disciplined -Hard work -Intelligent -CIS has little added value -Men’s study -Medical ‘island’ -Want to help people -Normal people -Open attitude -Solution oriented -Selectively interested -Socially empathetic -Status -Tunnel vision -Much knowledge -Feeling better |
|  | Prejudices about CIS students | -Always involved in communication -Different study attitude  -Wide interest -Broad knowledge -Understanding of business -Easy study -Motivation -Unknown future prospects -Fun study -Relaxed atmosphere -Students know each other well -Theoretical knowledge  -Unhealthy lifestyle |
|  | Self-judgement medicine students | -Thoughtful -High expectations -High workload -Critical -Medical ‘island’ -Unhealthy lifestyle -Perfectionist -Serious study attitude -Status -Difference in degree of commitment -Feeling better |
|  | Self-judgement CIS students | -Broad contribution -Broad knowledge -Ask for feedback -Lazy -Judge of character -Less added value -Unable to apply theory -Open attitude -Study for the fun  -Cooperate -Women’s study |
|  | Insight into one’s own expertise | -Medicine students’ own expertise -CIS students’ own expertise  -Insights in one’s own expertise |
|  | CIS students’ own expertise | -Understanding of importance of communication -Persuasion -Communication on different levels  -Targeted communication -Understanding of business -(business) organization and structures -Convince -Statistics -Text design -Tactical communication |
|  | Medicine students’ own expertise | -Doctor patient communication -Personal interaction -Collaboration -Multi-faceted communication |

Table 1. Themes, categories and subcategories extracted from the focus group interviews.
Please note: original data were in Dutch, we translated the data for readability reasons.
